# Corpus Linguistics as a Research Method in Nursing: A Practical Approach to Analysing Language Data

**DOI:** 10.1111/jan.16659

**Published:** 2025-01-02

**Authors:** Carmel Bond

**Affiliations:** ^1^ Department of Nursing and Midwifery, College of Health Wellbeing & Life Sciences Sheffield Hallam University Sheffield UK

**Keywords:** discourse analysis, qualitative approaches, research in practice, research methods

## Abstract

**Aim:**

To highlight the use of corpus linguistics for analysing language data and to provide a worked example of this approach in nursing research.

**Design:**

Methodology discussion paper.

**Methods:**

This paper introduces corpus linguistics as a distinct approach to undertaking qualitative research in nursing. Examples are provided to illustrate how corpus linguistics can be applied to explore contemporary concepts in healthcare.

**Conclusion:**

Corpus linguistics offers a structured, robust alternative to traditional qualitative analysis. When combined with critical social theory, it is ideal for exploring power dynamics and discourse, making it a valuable approach for nursing and healthcare related research.

**Implications for Nursing:**

Nurse researchers often work with large amounts of textual data. Corpus linguistics provides a rigorous framework for analysing such data sets, which can be used for various studies.

**Impact:**

Corpus linguistics analytics can enhance nursing research by uncovering language use patterns that can help generate knowledge to inform nursing practice and policy development.

**Patient or Public Contribution:**

None.

## 1 Introduction

Communication is essential to the delivery of patient‐centred care. Verbal and written communication is constantly taking place between nurses, patients and interdisciplinary teams, involving patient records, shift reports and care plans (Kwame and Petrucka 2021). These forms of communication, when captured and used as data for research, can be subjected to a detailed analysis using a corpus linguistic method (Brookes and Collins 2023). This involves examining linguistic patterns and structures in language using large collections of naturally occurring language or text data, known as corpora (McEnery and Brookes 2024). The method employs quantitative and qualitative techniques, offering a robust framework for analysing language use across diverse contexts. It is a semi‐automated method using a specific computer software (see Section 4 of this paper) that the researcher can upload data to. While the computer handles the data, the researcher drives the analytical process, choosing which words and phrases to search for and appraise. This approach enables the interpretation of word‐types, or sections of language use, leading to the identification of patterns of linguistic usage. The strength of this approach is the evidence‐based insights into linguistic phenomena that are generated, making it a powerful tool for addressing theoretical or applied research questions. Hence, corpus linguistics is a valuable methodological choice for the nurse researcher. However, despite its potential to systematically examine large amounts of data, corpus linguistics is not widely recognised and few nursing‐related studies have adopted this approach (Hunt and Brookes 2020). This paper aims to provide theoretical and practical knowledge for data analysis using corpus software, equipping nurse researchers with the necessary tools to conduct rigorous and meaningful textual analysis.

### 1.1 Background

Grounded in critical social theory, corpus linguistics provides a framework for examining how language reflects and constructs power dynamics, ideologies, and social inequalities (Stevenson 2021). Drawing from the work of theorists such as Fairclough (2013), this approach highlights how discourse shapes societal norms, identities, and power relations. Therefore, language becomes the foundation for both constructing and challenging social hierarchies.

Corpus linguistics offers a robust way to examine language use across different areas of nursing and healthcare research, allowing researchers to uncover hidden ideologies and power structures embedded in everyday communication. This perspective is particularly relevant in healthcare, where communication between professionals and patients (or nurses and other healthcare professionals) may reflect broader societal dynamics such as authority, as patient and professional autonomy. The alignment of corpus linguistics with critical social theory helps to expose these dynamics, thereby fostering a deeper understanding of how language operates within institutional settings like nursing (Huang 2024).

## 2 Qualitative Research in Nursing: Approaches and Rigour

The advancement of nursing depends on the development of a contemporary evidence base to drive innovation in practice, education, and policy (Connor et al. 2023). A central concern for many nurse researchers is understanding and interpreting individuals' healthcare experiences, which are essential for improving patient care and outcomes (Holloway and Galvin 2023). These types of research questions often require qualitative methods, as they are uniquely suited to capturing the depth, complexity, and nuance of human experiences, providing insights that quantitative approaches often overlook (Merriam and Tisdell 2015). Therefore, by undertaking qualitative research, nurse researchers can contribute knowledge that directly informs practice and policy, enhancing patient‐centred care.

There are various approaches to data analysis in qualitative research, all of which have advantages and critiques. While different qualitative methods have strengths and limitations, they are all underpinned by systematic practices that allow researchers to analyse data in a way that is transparent, replicable, and credible (Creswell and Poth 2016). For example, thematic analysis provides a flexible but systematic approach to identifying themes, narrative analysis explores meaning‐making through personal stories, and phenomenology delves into the essence of lived experience (Creswell and Creswell 2017).

To enhance the credibility and the trustworthiness of findings, qualitative researchers often use strategies such as reflexivity, triangulation and transparency (Ahmed 2024). However, inadequate data analysis is a primary reason for the rejection of qualitative research papers during the peer review process (Jackson and Bradbury‐Jones 2020). Researchers using thematic analysis (Braun and Clarke 2012) often misapply the analytical framework, leading to insufficient analysis or poorly developed themes (Finlay 2021). If the analysis is insufficient, the findings may be perceived as lacking credibility. Thus, the acceptance of knowledge derived from qualitative research is contingent on the rigour of the analysis, regardless of the findings' potential significance for clinical practice, education or policy.

In this context, corpus linguistics offers a distinct approach that can complement qualitative analysis in nursing research. A corpus‐based methodology employs techniques for analysing language that enhance the robustness of qualitative findings by offering quantitative support to qualitative insights (Brezina 2018). For example, using corpus software (outlined in Section 3 of this paper), researchers can generate a frequency list of all word‐types within the data, which can be analysed using computer software. Words (or sections of language use) can then be interpreted according to the research question or chosen theoretical focus for the study. This allows for a more objective interpretation of language use, thereby minimising biases that might typically be associated with human intuition (McEnery and Brookes 2024). Additionally, factors that are traditionally linked to quantitative research, such as reliability and external validity can be highlighted, as findings are transparent and reproducible (Chalmers 2013). Not only does this provide reassurance regarding the overall strength of the analytical process (as the findings can be verified or proven false), but it also enables patterns of linguistic usage to be identified within a reasonable timeframe (McEnery and Brookes 2024).

## 3 Language, Narratives, and Power in Nursing Research

The most common form of natural language occurs when individuals share stories about real‐life experiences, reflecting their beliefs and feelings on specific topics. In nursing research, these narratives – especially in the context of healthcare communication – erve as valuable data for understanding how people experience health, illness, medical procedures, and end‐of‐life issues, as well as their attitudes towards these phenomena (Riessman 2004). From a discourse theory perspective, these narratives do more than relay personal experiences, they construct and reflect broader social practices, power dynamics and identities. The linguistic construction of such accounts reveals the interaction between different textual features, allowing researchers to explore how language both shapes and is shaped by social contexts and healthcare environments.

Discourse and narrative theories are particularly relevant to corpus studies when working with naturally occurring language (Mautner 2022). For instance, Bond et al. (2018), in their study of compassion in nursing, drew on the work of sociological thinker and discourse scholar, Van Dijk (2006). Van Dijk's (2008) theory of ‘Discourse as Ideology’ highlights how discourse functions as a social practice that shapes cognition, identity and behaviour. External conceptualisations influence how individuals interpret their own experiences, which, in turn, guide social action (van Dijk 2008). Applying a corpus‐based analysis, Bond et al. (2018) illustrated how language users create mental models (in this case, based on media‐generated discourse of compassion) which thus influenced how compassion was being constructed as an individual inherent trait. This discourse placed the responsibility for the delivery of compassion on individual nurses, rather than at the organisational level. However, linguistic study often extends beyond individual experiences to examine how language reflects and sustains societal power structures. Ruth Wodak's (2015) work highlights that language is not neutral; it is shaped by those in power to maintain dominant ideologies and macrostructures, such as political institutions, economic systems and cultural norms. She argues that those in power control the production and dissemination of discourses, shaping societal narratives that reinforce their authority.

Wodak's (2015) discourse‐historical approach (DHA) reveals how discourses are historically embedded, making them appear natural or inevitable. The strategic employment of language legitimises power and social hierarchies, positioning individuals within society by assigning roles and identities based on prevailing norms. Roles, assigned through language, such as ‘patient’ or ‘doctor’, are shaped by dominant medical or political discourses (Lemke 2012). This process, known as subjectivation, involves individuals internalising these roles, thereby reproducing the power relations that govern them. Mohammed et al. (2021) present an example of the influence of subjectification using discourse analysis. They examined the impact of media and public discourse that referred to nurses as ‘heroes’ during the COVID‐19 pandemic. Their findings indicated that this discourse placed nurses at additional risk by normalising the use of items like bin bags as protective equipment. This was the result of issues related to the lack of, or poor supply of, the correct protective equipment (Press 2020). Mohammed et al. (2021) argued that framing nurses within a ‘heroes’ discourse made the increased risks they faced (as a result of insufficient protective equipment) more palatable to the general public. These examples illustrate how language both reflects and constructs social order through power dynamics, emphasising the importance of theory in corpus study design.

Corpus linguistics and critical discourse analysis are two distinct methodologies that often intersect in examining language use. Nartey and Mwinlaaru (2019) illustrate how critical discourse analysis seeks to expose underlying power structures, while corpus linguistics provides the quantitative grounding needed to systematically identify patterns across extensive datasets. Hence, by combining a critical focus (critical discourse analysis) with empirical rigour (corpus linguistics) researchers can create a robust analysis that both interprets and substantiates how language shapes, and is shaped by, social and institutional structures (Nartey and Mwinlaaru 2019).

As a standalone method, however, corpus linguistics can be invaluable in providing quantitative and objective insights that support the analysis of discursive structures and practices. This is because corpus linguistics is effective at revealing frequent linguistic structures that might otherwise be overlooked, particularly in large datasets (Biber 1993). Teubert and Krishnamurthy (2007) argue the method is effective for uncovering *“probabilities, trends, patterns and co‐occurrences of elements”* (p. 6), which helps to validate or challenge assumptions about discourse. McEnery and Hardie (2012) further emphasise the role of corpus linguistics in validating or refining linguistic theories, particularly when comparing the frequency and use of language across diverse corpora, which facilitates the questioning of language variation over time.

## 4 Building a Corpus: Text Types and Considerations

Building or designing a corpus typically involves collecting a wide variety of texts that are representative of language use and relevant to the research question(s) or objective(s). The texts used can be written or spoken; examples include:

- Social media posts such as Tweets, Facebook posts, Instagram captions and comments that reflect everyday language use in digital contexts.
- Blogs and online articles, for example, personal blogs, opinion pieces and articles from online platforms covering a wide range of topics and perspectives.
- Transcripts of spoken language, including conversations, interviews, speeches, debates, patient–nurse/doctor interactions and other forms of spoken discourse.
- Survey responses, such as open‐ended responses from questionnaires or surveys, capturing individual perspectives and opinions.
- Emails and text messages, and other digital communications in formal and informal settings, which capture how people write in everyday exchanges.

Depending on the goals of the research, each of the above examples can be used, whether to study variation in language, discourse practices or some other linguistic phenomenon. It is worth exploring the literature relevant to the research question to gauge which types of data might be most appropriate. These initial stages are necessary in preparation for the analysis. However, building a corpus from scratch is time‐consuming and requires meticulous attention to detail, as each individual comment (language use) must be manually extracted from the original source. Individual comments are then amalgamated to form the corpus. Afterwards, the whole corpus, no matter how large, can be instantly interrogated using concordance software (see Section 3 of this paper). Alternatively, an existing corpus may be available that can help reduce the research timeline. For example, Harvey et al. (2008) used an existing corpus of email communications from adolescents to understand the effectiveness of this type of health communication compared to face‐to‐face consultations.

### 4.1 Data Comparison With Existing Corpora

Data comparison is useful in corpus studies as this helps to determine whether the patterns observed within the data (study corpus) are reflective of language use in everyday settings, alongside the context being studied. The British National Corpus contains 100 million words collected from a wide range of genres, including fiction, non‐fiction, newspapers, academic texts and spoken conversations (BNC Consortium 2007). This is a valuable resource for research purposes and could be used to compare utilised with the study data to confirm ‘typical’ language use. For the nurse researcher, there may be relevant corpora available such as the ‘Nottingham Health Communication Corpus (NHCC)’, which consists of over half a million words and can be used to study the language of healthcare (illustrated in Baker, Brookes, and Evans 2019). This corpus focuses on how nurses and other healthcare professionals communicate with patients, deliver care, and manage interactions in clinical settings. There may be other data available, including de‐identified clinical records found with a specific health service. However, consideration must be given to the access requirements and costs involved in gaining access to specific corpora. Nonetheless, these resources can be extremely useful for busy clinicians who may wish to undertake research but are time‐limited.

## 5 Standard Corpus Tools for Observing Linguistic Patterns

The ability to observe patterns of language is made possible using corpus linguistic tools (Table 1). Some standard tools include frequency searches, keyword in context (KWIC) function, concordance lines and collocation analyses, which make it easier to see the structure of a text, and which words occur adjacent to one another. This then allows observations relating to ‘how’ language users use words in real‐life situations (Stubbs 2001).

**TABLE 1:** Descriptions of relevant terms (information drawn from Gillings, Mautner, and Baker 2023; Jones 2022).

Term(s)	Description/example
Discourse	Discourse is a broad term that refers to communication, either spoken or written, between people
Corpus (plural: corpora)	A large and structured collection of texts, usually in a digital format, used for linguistic research and analysis
Frequency list	A ranked list of words or linguistic units (such as words and similar word‐types) based on how often they occur in a given corpus
Keyword in context (KWIC)	A common tool used to display the occurrences of a particular word (“keyword”) within its surrounding context in a text or corpus. The KWIC analysis is useful for analysing how a word is used in different contexts, helping researchers to understand its meaning, usage patterns and collocations
Concordance line(s)	A concordance line is a short segment of text that displays occurrences of a specific word or phrase. The “keyword” is displayed at the centre of the concordance line and is shown within its immediate textual context
Collocation and collocation analysis	A set of words that occur together with a higher‐than‐random frequency. For example, suppose the KWIC function was used to analyse collocations of the word “commit” in a corpus. The analysis might reveal “commit” frequently occurs with words like “crime” and “murder.” These words would be considered strong collocates of “commit” because they co‐occur with it more often than would be expected by chance

**FIGURE 1:**
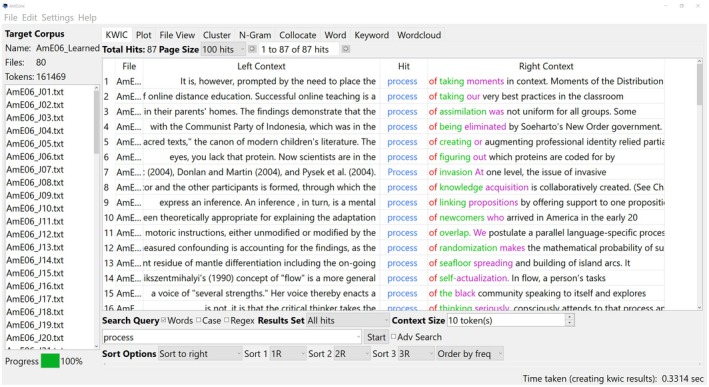
AntConc user interface—showing concordance lines with the keyword “process” in the centre.

Standard corpus tools are specialised software applications used to analyse and manipulate language data that are useful for gaining in‐depth insights. There are various software tools available, which differ in complexity and use, so the researcher can choose a programme to suit them and the type of corpus linguistic research they wish to perform—from basic text analysis to advanced, theory‐driven studies. For academic purposes, WordSmith Tools (Scott 2016) and AntConc (Anthony 2023) are both suitable for performing the types of analysis that this paper has described thus far. These tools assist researchers in exploring large collections of texts (corpora) by examining word frequencies, collocations, concordances, semantic patterns and more. Figure 1 is taken from Lawrence Anthony's Website and illustrates the user interface. The software is free to use and download.

### 5.1 Frequency Lists

It has been argued (Alsop et al. 2020) that the most common place to start is by conducting a frequency (‘word list’) search, although this is not possible without first building or designing the corpus. The stages involved in a corpus study, as discussed thus far, might begin with the chosen topic and theoretical perspective for the research. Hence, the design of the corpus is underpinned by these factors that must be determined before the analytical tools outlined in Table 1 can be used.

**FIGURE 2:**
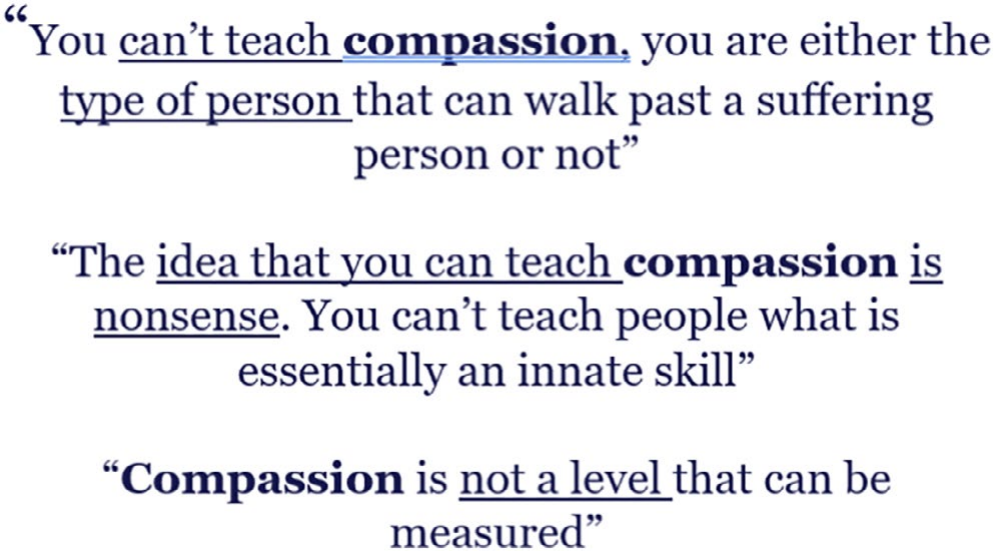
Extract from the analysis by Bond et al. (2018).

The keyword in context (KWIC) function permits patterns to be observed and words that were collocated^1^ to be made visible. Concordance lines tend to be driven by a frequency list. An example of a concordance line is shown in Figure 1, around the keyword ‘process’. In another example, Bond et al. (2018) observed patterns of collocated words around the keyword ‘compassion’ which was the topic of study (Figure 2), but the word compassion did not rank as high on the initial frequency list. Although compassion was not the most frequently referred word within the data, compassion was the focus of the study. Therefore, the researchers were driven to further interrogate the data, through a process of immersion, to expose patterns in language that might have otherwise been difficult to observe. For more detail, see Bond et al. (2018).

### 5.2 Limitations of Frequency Lists

Whilst frequency lists in corpus studies are useful, they also come with limitations that the researcher must acknowledge within their design and during the analysis. Frequency lists provide raw counts of word occurrences. However, they do not necessarily offer context for “how” words are used. This can make it difficult to understand the meaning, function, or nuances of words. For example, a high‐frequency word such as “bank” might refer to a financial institution or the side of a river. Hence, a frequency list alone cannot provide clarity of meaning, or indicate which type of meaning is common within the corpus.

If the corpus is a sample of English language, it would be typical to see words such as ‘the’, ‘and’, ‘of’ at the top of the list (Stubbs 2001). However, rare words that might be significant in certain contexts or within a different language or culture, may be overlooked due to their low frequency (Stubbs 2001). As such, it may be beneficial, depending upon the particular language or genre being appraised, for the researcher to have some degree of insight into the core vocabulary used (Brezina 2018). Another potential limitation is that the corpus may be criticised for not capturing the full spectrum of sociolinguistic variation, such as variation across social groups (e.g. age, gender, ethnicity). It is therefore worth planning who will undertake the analysis at the design stage. Whatever the case, the researcher/research team must look closely at their personal characteristics and critically appraise these within the study through an open and transparent process that recognises the limitations regarding representativeness. This is vital for contextualising the findings and addressing any potential bias in the data and subsequent reporting.

## 6 Representativeness in Corpus Study: Issues and Implications

When building or designing the corpus, ‘representativeness’ must be considered. This is important as it directly impacts issues such as validity and generalisability. Representativeness refers to ‘how well’ a corpus reflects the language use of a particular population, or the extent it echoes the context. However, what constitutes a ‘representative sample’ of language has been debated, as language use is inherently variable and context dependent (Biber 1993). McEnery and Hardie (2012) have argued that achieving accurate representativeness is impossible because it requires the researcher to have extensive knowledge of a complete population of texts, which is impractical to achieve.

The size of the corpus is also discussed, in terms of what is considered “truly” representative. Baker, Brookes, and Evans (2019) argue that a larger corpus is more representative because it captures a wider variety of language use, whereas others believe a bigger corpus leads to over‐sampling of certain types of language, which introduces bias (Egbert, Biber, and Gray 2022). There are other issues relating to the design of the corpus that are not covered here, such as theoretical versus practical concerns (discussed by O'Keeffe and McCarthy 2019), transparency, and justifying design choices (see Gries 2021). These authors raise questions regarding the nature of the corpus (whether it is a specialised form of language or general language use), the extent to which generalisations can be made using corpus‐based studies, and if the findings are transferrable to a different context (Kemp 2024; Deignan 2016). It is imperative that these issues are considered thoroughly at the design stage as this will enable the researcher to strengthen their arguments and justify design choices grounded in the goals of the research question.

### 6.1 Considering and Addressing Bias

Researchers must be aware of potential bias inherent in corpus creation (Hovy and Prabhumoye 2021). Studying certain genres, for example, may inadvertently emphasise particular social groups, ideologies or practices while privileging certain voices over others. To construct an unbiased corpus, careful consideration of text selection is essential. A representative sample helps prevent overrepresentation of specific linguistic features or perspectives, which could otherwise skew the analysis (Brookes and Collins 2023). Including language from diverse sources, for instance, texts covering various clinical interactions on a given topic ensures a broad spectrum of language use. Additionally, collecting texts from distinct levels or social strata, such as individual (patient stories, doctor‐patient dialogues), organisational (hospital policies, internal communications, professional guidelines), and government (regulations, health campaigns, legislative documents) captures a more holistic picture of how language is used at different levels of interaction (Reppen and Simpson‐Vlach 2019).

After the analysis, a strategy for cross‐checking findings, such as data comparison (Section 3.1), can be implemented to ensure consistency in language use or highlight atypical language patterns. Careful documentation of the text selection processes and transparent reporting of the corpus composition are essential to ensure that any biases are acknowledged (Egbert, Biber, and Gray 2022). Overall, this rigorous approach contributes to a balanced and credible analysis, enhancing the overall reliability of the research.

## 7 Using Corpus in Nursing: A Worked Example

In this final section, a worked example is provided, starting from designing the research question through to analysis and interpretation. Thereby illustrating how corpus linguistic analysis can be applied in nurse‐led research and aligned with discourse theory.

### 7.1 Step 1: Designing the Research Question

A mental health nurse may wish to explore how nurses use communication to foster therapeutic relationships with patients and how the language used might reflect broader institutional practices in mental health settings. This would involve several steps, beginning with setting the research question and deciding which theory to use.

The research question needs to be appropriate for corpus linguistics, therefore, it must focus on how specific language features are employed in therapeutic communication. The focus might be on how mental health nurses communicate with patients experiencing anxiety or depression. The addition of Wodak's (2015) discourse‐historical approach allows the focus to be on how nurses communicate, as well as the historical and institutional contexts influencing this communication. This will permit questions to be asked that facilitate an understanding of *‘how the language used by nurses is shaped by historical shifts in mental health care policies, institutional practices, and broader societal attitudes towards mental illness’*.

Therefore, the research question could be, *“How do mental health nurses use language to foster therapeutic relationships and support patients with anxiety or depression, and how do these practices reflect broader historical and institutional discourses in mental health care?”*

### 7.2 Step 2: Constructing the Corpus

After addressing ethical considerations, the corpus would likely consist of transcribed nurse–patient interactions in mental health settings. The researcher may also gather documents such as mental health policies, professional guidelines, educational information and archival materials from previous eras of mental health care (e.g. older textbooks or records from asylums or psychiatric institutions) that reflect institutional and historical shifts. This helps to illustrate how nurses' current language use may be shaped by past discourses. For example, it may reveal the transition from a ‘custodial care’ model, where patients were passive recipients (Raeburn, Bradshaw, and Cleary 2023), to a ‘recovery‐oriented’ model, where patients are active participants (Barker 2003). Another example might be ‘patient’ to ‘consumer’, which would be indicative of the rise of ‘consumerised care’ and the commodification of health. At this stage, it is important to be mindful of potential biases when selecting texts to construct the corpus.

### 7.3 Step 3: Analysing the Corpus

Standard corpus tools will enable the researcher to explore the language used (by mental health nurses) during patient interactions. Drawing from Wodak's (2015) theories of discourse, the analysis can focus on frequency and patterns of language, and how these patterns might reflect the historical context and power dynamics in mental health care.

A frequency and concordance analysis could examine how words like ‘control’ or ‘support’ are used. This might reveal how nurses frame patient autonomy today, compared to how this concept was expressed historically in older records/policy documents. For example, if ‘control’ is collocated with words like ‘patient’ and ‘decision’ in contemporary data but historically co‐occurred with word‐types like ‘nurse’ or ‘doctor’, this would support the researcher in suggesting that a shift in power dynamics has occurred over time.

A collocation analysis might assess how words like ‘patient’ and ‘diagnosis’ might be collocated with terms that reflect power relations, such as ‘permission’ or ‘order’. These patterns could be compared across time, meaning that the researcher's interpretation could be derived by tracking the discursive shift from the medical model of mental health to the recovery‐oriented model.

An additional keyword analysis may be useful for comparing the researcher developed corpus to historical corpora. Specific terms may be highlighted, which have increased or decreased in usage over time. For example, ‘asylum’ or ‘institution’ may have historically dominated, but today's discourse may focus more on the use of terms like ‘well‐being', ‘person‐centred care’, ‘mental health’, and ‘mental health issues', rather than ‘mental illnesses'. For more detailed guidance for analysing corpus, see Jones (2022).

## 8 Concluding Remarks

Corpus linguistics is a novel method, which few nursing‐related studies have adopted. However, a corpus‐based approach offers a structured and robust alternative to traditional qualitative analysis. When combined with critical social theory, the method is relevant for topics such as the influence of power on the nursing profession, or examining how communication might be shaped by societal and organisational discourses. Therefore making it a useful methodology in nurse‐led research.

## Conflicts of Interest

The author declares no conflicts of interest.

## Peer Review

The peer review history for this article is available at https://www.webofscience.com/api/gateway/wos/peer‐review/10.1111/jan.16659.

## Data Availability

Data sharing is not applicable to this article as no new data were created or analysed in this study.

## Endnotes

^1^
In corpus linguistics, a collocation is a series of words or terms that co‐occur more often than would be expected by chance. It is the syntagmatic attraction observed between words, phrases, or utterances within the corpus (Xiao 2015).
